# Supplementary material for: Neural and Synaptic Array Transceiver: A Brain-Inspired Computing Framework for Embedded Learning
Source: Front Neurosci. 2018 Aug 29;12:583. doi: 10.3389/fnins.2018.00583 (PMC6123384; doi:10.3389/fnins.2018.00583)
Supplement: Supplementary file 2 [file Data_Sheet_1.pdf]

SUPPLEMENTARY MATERIAL

Neural and Synaptic Array Transceiver: A Brain-Inspired Computing Framework for Embedded Learning

5.7 NSAT equations and Parameters

In this section we provide all the transition matrices ($\mathbf{A}$), the biases b, thresholds (θ) and reset values ($\mathbf{X}_r$) for all simulations (see Eq. (8)) in the main article.

5.7.1 NSAT Neuron Dynamics Support a Wide Variety of Neural Responses

We simulated six different neural responses (see Fig. 6). For every simulation we used one group of NSAT parameters (0) and no learning at all. Hence parameters for Fig. 6(a) are:

$$\mathbf{A}^0 = \begin{bmatrix} -4 & -16 & -16 & -16 \\ -16 & -7 & -16 & -16 \\ 0 & -16 & -2 & -16 \\ 0 & -16 & -16 & -6 \end{bmatrix}$$

$$\mathbf{sA}^0 = \begin{bmatrix} -1 & 1 & 1 & 1 \\ 1 & -1 & 1 & 1 \\ 1 & 1 & -1 & 1 \\ 1 & 1 & 1 & -1 \end{bmatrix}$$

$$\mathbf{b}^0 = \begin{bmatrix} -287 \\ -39 \\ 0 \\ 0 \end{bmatrix}$$

$$\mathbf{Xinit}^0 = \begin{bmatrix} -7000 \\ -5000 \\ 100 \\ 10 \end{bmatrix}$$

$$\mathbf{XresetOn}^0 = \begin{bmatrix} True \\ True \\ True \\ True \end{bmatrix}$$

$$\mathbf{Xreset}^0 = \begin{bmatrix} -7000 \\ -5000 \\ 0 \\ 0 \end{bmatrix}$$

Parameters for Fig. 6(b) are:

$$\mathbf{A}^0 = \begin{bmatrix} -4 & -8 & -16 & -16 \\ -16 & -7 & -16 & -16 \\ 0 & -16 & -2 & -16 \\ 0 & -16 & -16 & -6 \end{bmatrix}$$

$$\mathbf{sA}^0 = \begin{bmatrix} -1 & 1 & 1 & 1 \\ 1 & -1 & 1 & 1 \\ 1 & 1 & -1 & 1 \\ 1 & 1 & 1 & -1 \end{bmatrix}$$

$$\mathbf{b}^0 = \begin{bmatrix} -167 \\ -11 \\ 0 \\ 0 \end{bmatrix}$$

$$\mathbf{Xinit}^0 = \begin{bmatrix} -7000 \\ -5000 \\ 100 \\ 10 \end{bmatrix}$$

$$\mathbf{XresetOn}^0 = \begin{bmatrix} True \\ False \\ True \\ False \end{bmatrix}$$

$$\mathbf{Xreset}^0 = \begin{bmatrix} -7000 \\ 0 \\ 500 \\ 0 \end{bmatrix}$$

Parameters for Fig. 6(c) are:

$$\mathbf{A}^0 = \begin{bmatrix} -4 & -16 & -16 & -16 \\ -16 & -7 & -16 & -16 \\ 0 & -16 & -2 & -16 \\ 0 & -16 & -16 & -6 \end{bmatrix}$$

$$\mathbf{sA}^0 = \begin{bmatrix} -1 & 1 & 1 & 1 \\ 1 & -1 & 1 & 1 \\ 1 & 1 & -1 & 1 \\ 1 & 1 & 1 & -1 \end{bmatrix}$$

$$\mathbf{b}^0 = \begin{bmatrix} -287 \\ -39 \\ 0 \\ 0 \end{bmatrix}$$

$$\mathbf{Xinit}^0 = \begin{bmatrix} -7000 \\ -5000 \\ 100 \\ 10 \end{bmatrix}$$

$$\mathbf{XresetOn}^0 = \begin{bmatrix} True \\ True \\ True \\ True \end{bmatrix}$$

$$\mathbf{Xreset}^0 = \begin{bmatrix} -7000 \\ -5000 \\ 0 \\ 0 \end{bmatrix}$$

Parameters for Fig. 6(d):

$$\mathbf{A}^0 = \begin{bmatrix} -4 & -8 & -16 & -16 \\ -16 & -7 & -16 & -16 \\ 0 & -16 & -2 & -16 \\ 0 & -16 & -16 & -6 \end{bmatrix}$$

$$\mathbf{sA}^0 = \begin{bmatrix} -1 & 1 & 1 & 1 \\ 1 & -1 & 1 & 1 \\ 1 & 1 & -1 & 1 \\ 1 & 1 & 1 & -1 \end{bmatrix}$$

$$\mathbf{b}^0 = \begin{bmatrix} -194 \\ -11 \\ 0 \\ 0 \end{bmatrix}$$

$$\mathbf{Xinit}^0 = \begin{bmatrix} -3000 \\ -3000 \\ 100 \\ 10 \end{bmatrix}$$

$$\mathbf{XresetOn}^0 = \begin{bmatrix} True \\ False \\ True \\ True \end{bmatrix}$$

$$\mathbf{Xreset}^0 = \begin{bmatrix} -7000 \\ -6000 \\ 0 \\ 0 \end{bmatrix}$$

Parameters for Fig. 6(e):

$$\mathbf{A}^0 = \begin{bmatrix} -4 & -8 & -16 & -16 \\ -16 & -7 & -16 & -16 \\ 0 & -16 & -2 & -16 \\ 0 & -16 & -16 & -6 \end{bmatrix}.$$

$$\mathbf{sA}^0 = \begin{bmatrix} -1 & 1 & 1 & 1 \\ 1 & -1 & 1 & 1 \\ 1 & 1 & -1 & 1 \\ 1 & 1 & 1 & -1 \end{bmatrix}$$

$$\mathbf{b}^0 = \begin{bmatrix} -250 \\ -10 \\ 0 \\ 0 \end{bmatrix}$$

$$\mathbf{Xinit}^0 = \begin{bmatrix} -7000 \\ -5000 \\ 100 \\ 10 \end{bmatrix}$$

$$\mathbf{XresetOn}^0 = \begin{bmatrix} True \\ False \\ True \\ False \end{bmatrix}$$

$$\mathbf{Xreset}^0 = \begin{bmatrix} -7000 \\ -6000 \\ 0 \\ 0 \end{bmatrix}$$

Parameters for Fig. 6(f):

$$\mathbf{A}^0 = \begin{bmatrix} -4 & -8 & -16 & -16 \\ -16 & -7 & -16 & -16 \\ 0 & -16 & -2 & -16 \\ 0 & -16 & -16 & -6 \end{bmatrix}$$

$$\mathbf{sA}^0 = \begin{bmatrix} -1 & 1 & 1 & 1 \\ 1 & -1 & 1 & 1 \\ 1 & 1 & -1 & 1 \\ 1 & 1 & 1 & -1 \end{bmatrix}$$

$$\mathbf{b}^0 = \begin{bmatrix} -194 \\ -11 \\ 0 \\ 0 \end{bmatrix}$$

$$\mathbf{Xinit}^0 = \begin{bmatrix} -7000 \\ -5000 \\ 100 \\ 10 \end{bmatrix}$$

$$\mathbf{XresetOn}^0 = \begin{bmatrix} True \\ False \\ True \\ False \end{bmatrix}$$

$$\mathbf{Xreset}^0 = \begin{bmatrix} -7000 \\ -6000 \\ 1000 \\ 0 \end{bmatrix}$$

Amari's Neural Fields

In the NSAT implementation of Amari's neural fields we used only one NSAT parameters group (0) and no learning parameters since there is no learning in the simulations we ran. Therefore, all the parameters we used for simulating the neural fields in the NSAT are given below.

$$\mathbf{A}^0 = \begin{bmatrix} -2 & -16 & -16 & -16 \\ -16 & -16 & -16 & -16 \\ -16 & -16 & -16 & -16 \\ -16 & -16 & -16 & -16 \end{bmatrix} \quad \mathbf{sA}^0 = \begin{bmatrix} -1 & 1 & 1 & 1 \\ 1 & 1 & 1 & 1 \\ 1 & 1 & 1 & 1 \\ 1 & 1 & 1 & 1 \end{bmatrix} \quad \mathbf{b}^0 = \begin{bmatrix} -5 \\ 0 \\ 0 \\ 0 \end{bmatrix}$$

$$\mathbf{Xinit}^0 = \begin{bmatrix} 0 \\ 0 \\ 0 \\ 0 \end{bmatrix} \quad \mathbf{XresetOn}^0 = \begin{bmatrix} False \\ False \\ False \\ False \end{bmatrix} \quad \mathbf{Xreset}^0 = \begin{bmatrix} 0 \\ 32767 \\ 32767 \\ 32767 \end{bmatrix}$$

Supervised Event-based Deep Learning

Three neuron groups were used and denoted as superscripts: hidden neurons (H), output neurons (O) and error neurons (E).

$$\mathbf{A}^H = \begin{bmatrix} -7 & -16 \\ -16 & -6 \end{bmatrix} \quad \mathbf{A}^E = \begin{bmatrix} -16 & -16 \\ -16 & -16 \end{bmatrix} \quad \mathbf{A}^O = \begin{bmatrix} -7 & -16 \\ -16 & -6 \end{bmatrix}$$

$$\mathbf{sA}^H = \begin{bmatrix} -1 & 1 \\ 1 & -1 \end{bmatrix} \quad \mathbf{sA}^E = \begin{bmatrix} -1 & 1 \\ 1 & -1 \end{bmatrix} \quad \mathbf{sA}^O = \begin{bmatrix} -1 & -1 \\ -1 & -1 \end{bmatrix}$$

$$\mathbf{hiac} = \begin{bmatrix} -7 \\ 0 \\ 0 \end{bmatrix}$$

$$\mathbf{prob\_syn}^H = \begin{bmatrix} 9 \\ 15 \end{bmatrix} \quad \mathbf{prob\_syn}^E = \begin{bmatrix} 15 \\ 15 \end{bmatrix} \quad \mathbf{prob\_syn}^O = \begin{bmatrix} 9 \\ 15 \end{bmatrix}$$

$$\mathbf{Wgain}^H = \begin{bmatrix} 3 \\ 4 \end{bmatrix} \quad \mathbf{Wgain}^E = \begin{bmatrix} 4 \\ 4 \end{bmatrix} \quad \mathbf{Wgain}^O = \begin{bmatrix} 3 \\ 4 \end{bmatrix}$$

$$\mathbf{XresetOn}^{H} = \begin{bmatrix} False \\ False \end{bmatrix} \quad \mathbf{XresetOn}^{E} = \begin{bmatrix} False \\ False \end{bmatrix} \quad \mathbf{XresetOn}^{O} = \begin{bmatrix} False \\ False \end{bmatrix}$$

$$\mathbf{XspikeIncrVal}^{H} = \begin{bmatrix} 0 \\ 0 \end{bmatrix} \quad \mathbf{XspikeIncrVal}^{E} = \begin{bmatrix} -1025 \\ 0 \end{bmatrix} \quad \mathbf{XspikeIncrVal}^{O} = \begin{bmatrix} 0 \\ 0 \end{bmatrix}$$

$$\mathbf{Xthlo}^{H} = \begin{bmatrix} -32767 \\ -32767 \end{bmatrix} \quad \mathbf{Xthlo}^{E} = \begin{bmatrix} 0 \\ 0 \end{bmatrix} \quad \mathbf{Xthlo}^{O} = \begin{bmatrix} -32767 \\ -32767 \end{bmatrix}$$

Unsupervised Representation Learning

For the NSAT implementation of the eRBM and eRBMhp we used two different NSAT parameters groups and two different learning parameters groups, one for the visible units (V) and another for the hidden ones. All the parameters are given in the following equations.

$$\mathbf{A}^{V} = \begin{bmatrix} -3 & -16 & -16 & -16 \\ 8 & -5 & -16 & -16 \\ -16 & -16 & -16 & -16 \\ 8 & -16 & -16 & -5 \end{bmatrix} \quad \mathbf{A}^{H} = \begin{bmatrix} -3 & -16 & -16 & -16 \\ 8 & -5 & -16 & -16 \\ -16 & -16 & -16 & -16 \\ 8 & -16 & -16 & -5 \end{bmatrix}$$

$$\mathbf{sA}^{V} = \begin{bmatrix} -1 & 1 & 1 & 1 \\ 1 & -1 & 1 & 1 \\ 1 & 1 & -1 & 1 \\ 1 & 1 & 1 & -1 \end{bmatrix} \quad \mathbf{sA}^{H} = \begin{bmatrix} -1 & 1 & 1 & 1 \\ 1 & -1 & 1 & 1 \\ 1 & 1 & -1 & 1 \\ 1 & 1 & 1 & -1 \end{bmatrix}$$

$$\mathbf{b}^{V} = \begin{bmatrix} -6000 \\ 0 \\ 0 \\ 0 \end{bmatrix} \quad \mathbf{b}^{H} = \begin{bmatrix} -9500 \\ 0 \\ 0 \\ 0 \end{bmatrix}$$

$$\mathbf{hiac}^{V} = \begin{bmatrix} 0 \\ 0 \\ 0 \end{bmatrix} \quad \mathbf{hiac}^{H} = \begin{bmatrix} 1 \\ 0 \\ -1 \end{bmatrix}$$

$$\mathbf{hica}^{V} = \begin{bmatrix} 0 \\ 0 \\ 0 \end{bmatrix} \quad \mathbf{hica}^{H} = \begin{bmatrix} 1 \\ 0 \\ -1 \end{bmatrix}$$

$$\mathbf{probsyn}^{V} = \begin{bmatrix} 15 \\ 7 \\ 15 \\ 15 \end{bmatrix} \quad \mathbf{probsyn}^{H} = \begin{bmatrix} 15 \\ 7 \\ 15 \\ 15 \end{bmatrix}$$

$$\mathbf{siac}^{V} = \begin{bmatrix} 1 \\ 1 \\ 1 \end{bmatrix} \quad \mathbf{siac}^{H} = \begin{bmatrix} -1 \\ -1 \\ -1 \end{bmatrix}$$

$$\mathbf{tac}^V = \begin{bmatrix} -35 \\ -35 \end{bmatrix} \quad \mathbf{tac}^H = \begin{bmatrix} -16 \\ -36 \end{bmatrix}$$

$$\mathbf{tca}^V = \begin{bmatrix} 35 \\ 35 \end{bmatrix} \quad \mathbf{tca}^H = \begin{bmatrix} 16 \\ 36 \end{bmatrix}$$

$$\mathbf{Wgain}^V = \begin{bmatrix} 2 \\ 1 \\ 0 \\ 0 \end{bmatrix} \quad \mathbf{Wgain}^H = \begin{bmatrix} 2 \\ 1 \\ 0 \\ 5 \end{bmatrix}$$

$$\mathbf{Xreset}^V = \begin{bmatrix} 0 \\ 32767 \\ 32767 \\ 32767 \end{bmatrix} \quad \mathbf{Xreset}^H = \begin{bmatrix} 0 \\ 32767 \\ 32767 \\ 32767 \end{bmatrix}$$

$$\mathbf{Xthlo}^V = \begin{bmatrix} -32768 \\ -32768 \\ -1 \\ -32768 \end{bmatrix} \quad \mathbf{Xthlo}^H = \begin{bmatrix} -32768 \\ -32768 \\ -1 \\ -32768 \end{bmatrix}$$

$$\mathbf{Xthup}^V = \begin{bmatrix} 32767 \\ 32767 \\ 1 \\ 32767 \end{bmatrix} \quad \mathbf{Xthup}^H = \begin{bmatrix} 32767 \\ 32767 \\ 1 \\ 32767 \end{bmatrix}$$

Unsupervised Learning in Spike Trains

The parameters $b_2 = -V_{lth} = -1216$, $b_3 = \eta_h = 5$, $w^\gamma = 1024$, $a_{31} = -\eta_h/\bar{\text{C}}\text{a} = -9$ and $a_{00} = -4$, $a_{11} = -8$ are chosen to appropriately replicate the corresponding state decay time constants and couplings.

$$\mathbf{A}^0 = \begin{bmatrix} -4 & 0 & -16 & -16 \\ -16 & 0 & -16 & 0 \\ -16 & -16 & -8 & -9 \\ -16 & -16 & -16 & 0 \end{bmatrix}$$

$$\mathbf{sA}^0 = \begin{bmatrix} -1 & 1 & 1 & 1 \\ 1 & -1 & 1 & 1 \\ 1 & 1 & -1 & -1 \\ 1 & 1 & 1 & -1 \end{bmatrix}$$

$$\mathbf{b}^0 = \begin{bmatrix} 0 \\ -1216 \\ 0 \\ 5 \end{bmatrix}$$

$$\mathbf{hiac}^{0} = \begin{bmatrix} 1 \\ 4 \\ 0 \end{bmatrix}$$

$$\mathbf{hica}^{\mathbf{0}} = \begin{bmatrix} 2 \\ 0 \\ -2 \end{bmatrix}$$

$$\mathbf{tac}^{0} = \begin{bmatrix} 16 \\ 36 \end{bmatrix}$$

$$\mathbf{XspikeIncrVal}^{0} = \begin{bmatrix} 0 \\ 0 \\ 1024 \\ 0 \end{bmatrix}$$

$$\mathbf{Xreset}^{0} = \begin{bmatrix} 0 \\ 32767 \\ 32767 \\ 32767 \end{bmatrix}$$

$$\mathbf{Xthlo}^{0} = \begin{bmatrix} 0 \\ -2 \\ -32767 \\ -32767 \end{bmatrix}$$

$$\mathbf{Xthup}^{0} = \begin{bmatrix} 32767 \\ 8 \\ 32767 \\ 32767 \end{bmatrix}$$
