# Supplementary material for: Neural and Synaptic Array Transceiver: A Brain-Inspired Computing Framework for Embedded Learning
Source: Front Neurosci. 2018 Aug 29;12:583. doi: 10.3389/fnins.2018.00583 (PMC6123384; doi:10.3389/fnins.2018.00583)
Supplement: Supplementary file 3 [file Data_Sheet_2.pdf]

5 APPENDIX

5.1 Notation and Abbreviations

Symbols	Description
$\mathbf{A}$	Transition matrix
$\mathbf{b}$	Bias term
$\mathrm{x}[t]$	Neural state
$\boldsymbol{\epsilon}[t]$	STDP eligibility function
$K(\cdot)$	STDP kernel function
$\mathbf{W}$	Synaptic weights
$\boldsymbol{\theta}$	Threshold
$\boldsymbol{\eta}$	Additive noise
Ξ	Multiplicative noise (Bernoulli distribution)
$\mathbf{X}_r$	Reset value
$\mathrm{s}[t]$	Spike train
$>>$	Right bit shift
$\circ$	Hadamard product
$\diamond$	Bit-shift multiplication operation
$\circleddiamond$	Zero-rounding bit shift operation

Table 1. Notation

Abbreviation	Description
NSAT	Neural and Synaptic Transceiver Array
FPGA	Field-programmable Gate Array
DED	Differed Event-driven
AER	Address Event Representation
AON	Always ON
RLE	Run Length Encode
STDP	Spike-timing Dependent Plasticity
LTP	Long-term Potentiation
LTD	Long-term Depression
DSP	Digital Signal Processing
RTL	Register Transfer Level
SNN	Spiking Neural Network
ASIC	Application-specific integrated circuit
MNN	Mihalas-Niebur Neuron
DoG	Difference of Gaussians
SynOp	Synaptic Operations
MAC	Multiplication Accumulation
GPU	Graphics Processing Unit
eCD	event-based Contrastive Divergence
S2M	Synaptic Sampling Machine
eRBP	event-based Random Back-propagation
RBM	Restricted Boltzmann Machine
eRBM	event-based Restricted Boltzmann Machine
eRBMhp	event-based Restricted Boltzmann Machine high precision

Table 2. Abbreviations

5.2 Algorithmic description of bit-shift operators

Algorithm 2 Zero-rounding bit shift operation (◈)

```
function a ◈ x
    y = a ⋄ x
    if y ≠ 0 and a = 0 then
        return sign(−y)
    else
        return a
    end if
end function
```

Algorithm 3 Bit shift multiplication operation (⋄)

```
function a ⋄ x
    if a ≥ 0 then
        return x ≪ a
    else if a < 0 then
        return sign(x)(|x| ≫ −a)
    end if
end function
```

5.3 Software Implementation Details

In order to demonstrate the capabilities of the proposed NSAT framework, we implemented a multi-thread software simulator in the C programming language called cNSAT. The software has been designed to accommodate foreseeable specifications imposed by the hardware, and thus all operations use 16-bit integer (fixed-point) and binary arithmetics (no multiplications) as described in the Difference Equations of NSAT Framework section.

5.3.1 Data structures

Each thread is implemented as a large data structure that contains all the necessary data structures for implementing NSAT. The most significant data structure is the one that implements the neuron. Each neuron unit structure carries all the parameters necessary for integrating the neuron's dynamics and performing learning (Fig. 11). We distinguish the neurons into two main categories, external neurons and internal (NSAT) neurons. External neurons have plastic (adjustable) post–synaptic weights and STDP counters, but no dynamics. Internal neuron dynamics follow Eq. (6). Every neuron consists of a synaptic tree implemented as a linked list containing all the post–synaptic weights and the id number of the post–synaptic neurons. Only internal neurons have access to the `NSAT params structure` and to the `Learning params structure`. In addition, a state data structure is added to the internal neurons for keeping track of the dynamics (state of the neuron).

Every thread data structure has as members the neuron's data structure (internal and external), the spike event lists, some temporary variables that are used for storing results regarding NSAT dynamics, variables that gather statistics, monitor flags, filenames strings for on–line storing of spike events or other variables (such as neuron states and synaptic weights), and finally shared to neuron parameters such as number of neurons (internal and external) within a thread, and other parameters (see Parameters paragraph below).

5.3.2 Random Number Generator

The random number generator (RNG) is implemented in two different ways. First, we used the PCG RNG library (O'Neill, 2014) [3] to implement a uniform random number generator with long period.

[3] `http://www.pcg-random.org/`

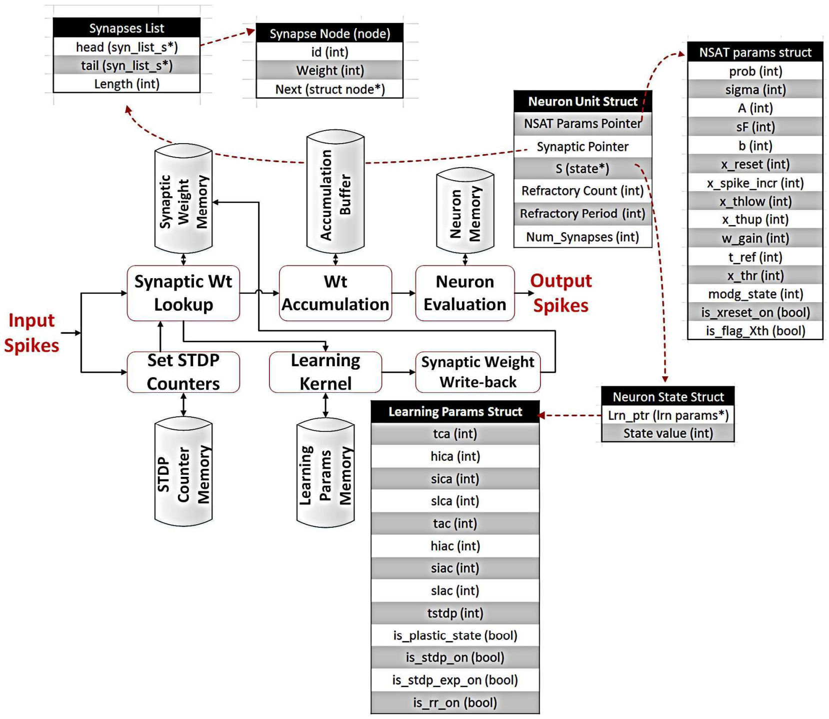

Figure 11. Schematic representation of NSAT data structures and information flow. The neuron structure is the main component of the NSAT software simulator. The neuron's dynamics, learning and state parameters data structures are shown. Red and black arrows represent pointers and information flow, respectively. See the text for more information regarding the parameters and information flow.

Based on the PCG library we implemented a Box–Muller (Box et al., 1958) transformation in order to acquire normal distributions for the additive noise of the NSAT framework.

The second implementation is used for simulating hardware implementations of the NSAT framework. In order to reliably generate uniformly distributed random numbers in a hardware implementation, we used a linear feedback shift register (LFSR) in combination with a cellular automata shift register (CASR) (Tkacik, 2002). Such types of RNGs are suitable for hardware devices and provide a robust and reliable random number generator. This implementation is used for bit accurate simulations of future NSAT hardware implementations.

5.3.3 Parameters

NSAT framework parameters can be split into three main classes. The first one contains global parameters related to the entire simulation and the configuration of the NSAT framework. The second class includes parameters for neurons dynamics and for the learning process. Figure 11 shows the neuron's and learning parameters (`NSAT_params` and `Learning_params` structures, respectively). The third class contains parameters local to each thread.

Parameters of the first class are the number of simulation time steps (or ticks), the total number of threads, the seeds and the initial sequences for the random number generators, a flag (Boolean variable) that indicates which of the two random number generators is used, a learning flag (Boolean variable) that enables learning, and the synaptic strengths boundary control flag (Boolean variable) that enables a synaptic weight range check to more closely match hardware implementations.

The second class of parameters, the neuron parameters (refer to `NSAT params struct`), includes the state transition matrix $\mathbf{A}$, the constant current or bias $\mathbf{b}$, and $\mathbf{sA}$ matrix which contains the signs of matrix $\mathbf{A}$. $\boldsymbol{\sigma}$ is the variance for the additive normal distributed noise and $\mathbf{p}$ is the blank-out probability (corresponding to Ξ in Eq. (8)). In addition, it contains the spike threshold ($\boldsymbol{\theta}$), the reset value ($\mathbf{X_r}$), the

upper ($\mathbf{X}_{\text{up}}$) and the lower boundaries ($\mathbf{X}_{\text{low}}$) for each neural state component. The latter two constants define the range of permitted values for each state component. The spike increment value increases or decreases the after-spike state component value instead of resetting it. A Boolean parameter enables or disables the reset of a neuron. An optional parameter permits variable firing threshold, whereby component x_1 is used as firing threshold for the neuron. An integer parameter defines which state component is assigned as plasticity modulator. Finally, another parameter sets the synaptic weights gains.

The third group consists of learning parameters. Each neural state component has its own synaptic plasticity parameters (see Fig. 11), enabled by a single flag. The rest of the learning parameters define the STDP kernel function ($K(\cdot)$ in Eq. (11)). The STDP kernel function is either a piecewise linear function or a piecewise exponential one that can approximate the classical STDP exponential curve or other kernel functions. The approximation uses either three linear segments for which we define the length, the height (level) and the sign or three exponential-like segments for which we define the length, the height, the sign and the slope, thus we have eight parameters that define the kernel function (four for the causal part and four for the acausal one).

Fig. 12(c) illustrates a realization of NSAT approximated STDP kernel function. `tca` (`tac`) controls the length (time dimension), `hica` (`hiac`) controls the amplitude (height), `sica` (`siac`) defines the sign for the causal (acausal) part, and `slca` (`slac`) characterizes the slope of the exponential approximation. On a given thread, different types of neurons and synapses can be defined by assigning them to separate parameter groups.

Finally, the third parameter group concerns the core configuration. Each parameter group specifies the number of internal and external units within a thread, states configurations per thread and in addition some extra parameters for the use of temporary variables necessary in simulations.

5.3.4 Python Interface

In order to facilitate the use of the software simulator (and the hardware later on) we developed a high-level interface in Python. The Python Interface (pyNSAT from now on) is based on Numpy, Matplotlib, Scipy, pyNCS and Scikit-learn Python packages. The pyNCS (Stefanini et al., 2014) is used for generating spike trains, read and write data from/to files and it provides proper tools for data analysis and visualization of simulations results.

Figure 13 illustrates an example of pyNSAT script simulating a neuron with four state components ($x_i, i = 0 \ldots 3$). The script mainly consists of five parts. First, we instantiate the configuration class. This class contains all the necessary methods to configure the simulation architecture and define the global parameters for the simulation, such as the total number of threads (or cores) to be used, number of neurons per thread, number of state components per neuron per thread, number of input neurons per thread and the simulation time (in ticks), Fig. 13(a). NSAT model parameters such as the matrix $\mathbf{A}$ of the NSAT dynamics, the biases $\mathbf{b}$ and many other parameters regarding the dynamics of the neuron and the model are defined as shown in Fig. 13(b). The next step is to define the synaptic connectivity (the architecture of the network), Fig. 13(c). Fig. 13(d) illustrates how the pyNSAT writer class is invoked for writing all the binary files containing the parameters that the C library will use to execute the simulation. Finally, we call the C NSAT library and execute the simulation (see Fig. 13(e)).

The shown example is a single neuron with adaptive threshold (more details regarding this neural model are given in Mihalas–Niebur Neuron paragraph in Results section). After simulating the model, we can visualize the results (see the right bottom panel in Fig. 13. The first row shows the membrane potential (state component $x_0[t]$, blue line) of the neuron, the second row indicates the adaptive threshold ($x_1[t]$, black line), and the third and fourth rows are two internal currents ($x_2[t]$ and $x_3[t]$, magenta and cyan colors), respectively.

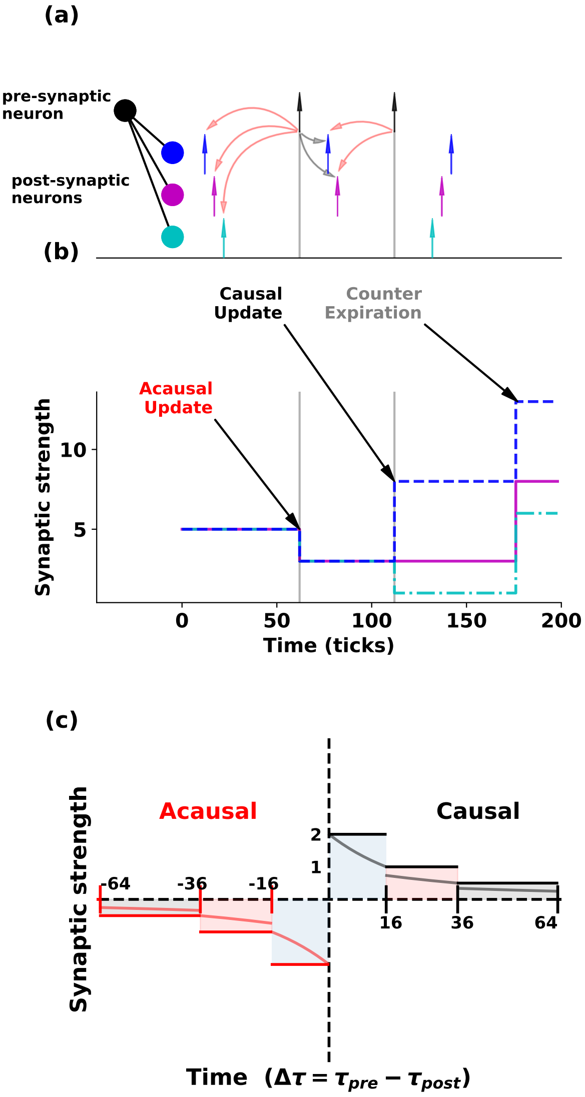

Figure 12. NSAT STDP learning rule. An actual simulation of a neuron (black dot, black spikes) connected to three post-synaptic ones (blue, magenta, cyan). **(a)** A pre–synaptic neuron (black node) projects to three post-synaptic neurons (blue, magenta and cyan nodes). Three spikes are emitted by the post-synaptic neurons (corresponding colored arrows) and then a spike is fired by the pre–synaptic neuron. Then an acausal update takes place since the post-synaptic spikes triggered within the acausal STDP time-window. Most recent post-synaptic spikes cause a causal update within the temporal limit defined by the causal STDP time-window. The light-gray lines indicate the pre-synaptic neuron's spike time, the red and black arrows illustrate the acausal and causal updates, respectively. **(b)** Temporal evolution of the post-synaptic weights. The acausal and causal updates are aligned with panel's (a) spikes. The gray vertical lines indicate the pre-synaptic spikes. Notice the latest weights update at 187 ticks. These causal updates are due to the expiration of the pre-synaptic neuron's counter (pre-synaptic neuron does not fire a spike at that time). **(c)** The STDP kernel function (linear and exponential approximations) used in this simulation (only the linear part). Black and red colors indicate the causal (positive) and the acausal (negative) parts of the STDP kernel function, respectively. Darker–colored lines illustrate the linear approximated STDP curves, and lighter–colored ones the exponential approximation (both types are supported by the NSAT framework).

5.3.5 Simulation Details

The software simulator uses a simulator implemented in the C Programming Language. All the source code used in this work are distributed under the GPL v3.0 License and are available on-line (https://github.com/nmi-lab/NSAT). All simulation parameters are provided in the source code accompanying this work and in the Supplementary Information. The Python interface for the NSAT framework has been written for Python 2.7.

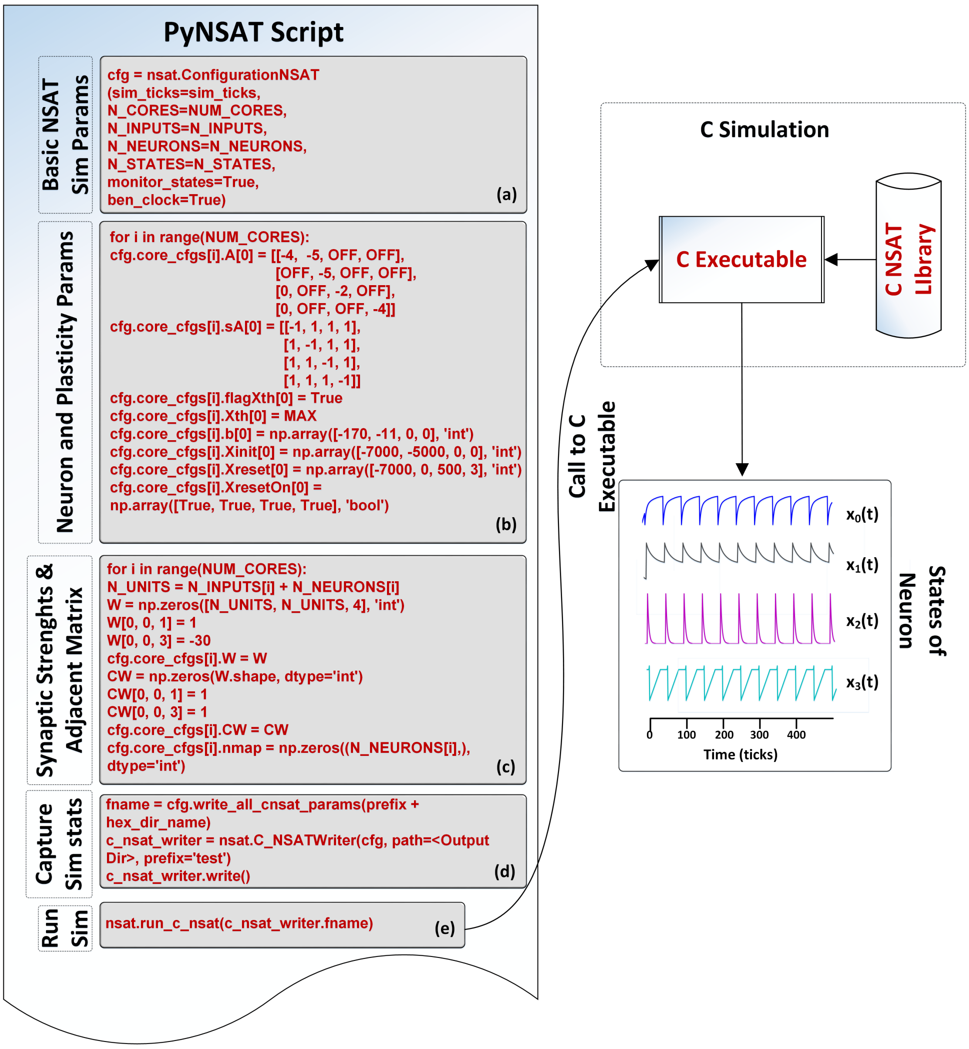

Figure 13. PyNSAT Example. Source code snippets for creating a simple simulation of a single neuron with four state components ($x_i, i = 0, \ldots, 3$). The Python script (**a**) instantiates the main configuration class, (**b**) sets neural dynamics, (**c**) defines the architecture of the network (synaptic connections), (**d**) writes all the parameters files and finally (**e**) calls the C library for running the simulation. The results of the simulation can be easily visualized using Python's packages.

5.4 Amari's Neural Fields Simulation

In order to numerically solve Eq. (15), we temporally discretize it using the Forward Euler method and thus we have,

$$u_i[t+1] = u_i[t] + \frac{\mathrm{d}t}{\tau}\Big(-u_i[t] + I_i^{\text{ext}} + h_i + dx \sum_{j=1}^{k} w_{ij} f(u_j[t])\Big), \tag{23}$$

where i is the spatial discrete node (unit or neuron), dt is the Euler's method time step and dx is the spatial discretization step.

As input to the neural field we use a Gaussian function with variance of 0.3 (black dashed line in Fig. 14(a)). At the end of the simulation the neural field has converged to its stable solution which is a "bump", as expected with a DoG kernel function (Fig. 14(a), red line). Figure 14(b) depicts the temporal evolution of numerical integration of Eq. (15) using the following parameters: $K_e = 1.5\, K_i = 0.75$, $\sigma_e = 0.1$ and $\sigma_i = 1.0$. The integral is defined in $\Omega = [0, 1]$ and we simulate for 50 seconds. After

about 7 seconds (200 simulation steps) the numerical solution converges to a fixed point (see (Amari, 1977) for more details).

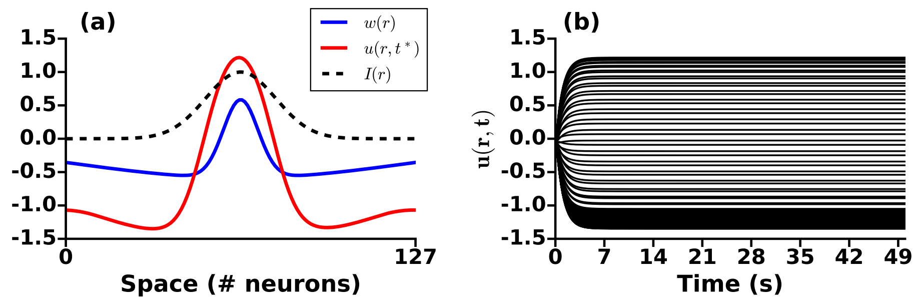

Figure 14. Amari's Neural Field of Infinite Precision. A numerical simulation of Eq. (23). In (**a**) Blue and red solid lines indicate the lateral connectivity kernel ($w(r)$) and a solution $u(r, t^*)$ for a fixed time step t^*, respectively. The black dashed line displays the input I_{ext} to the neural field Eq. (23). In (**b**) is illustrated the temporal numerical evolution of Eq. (23) for every spatial unit i. It is apparent that after about 7 seconds the system has reached its equilibrium point.

5.5 Supervised Event-based Learning

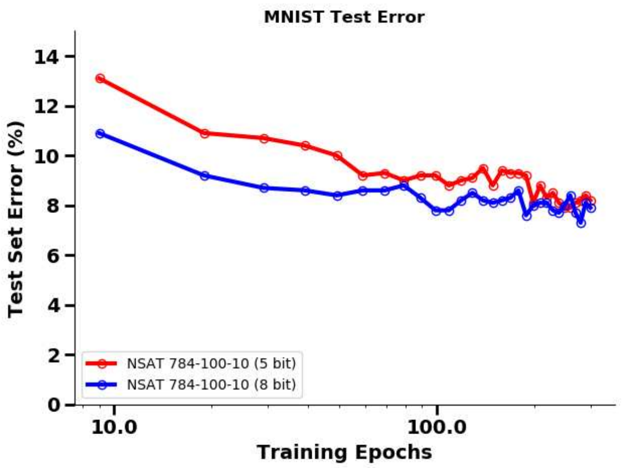

Figure 15. 8-bit and 5-bit synaptic precision. The red curve illustrates the test error of the feed-forward network described in section 3.3 trained with 5 bit synaptic precision. The blue curve indicates the same network trained with 8-bit synaptic precision. It is apparent that at the initial stages of learning the higher precision (8-bits) leads to a smaller error than the 5-bit precision. However, at the later states of learning both networks express similar behavior having similar test errors.

5.6 Unsupervised Representation Learning

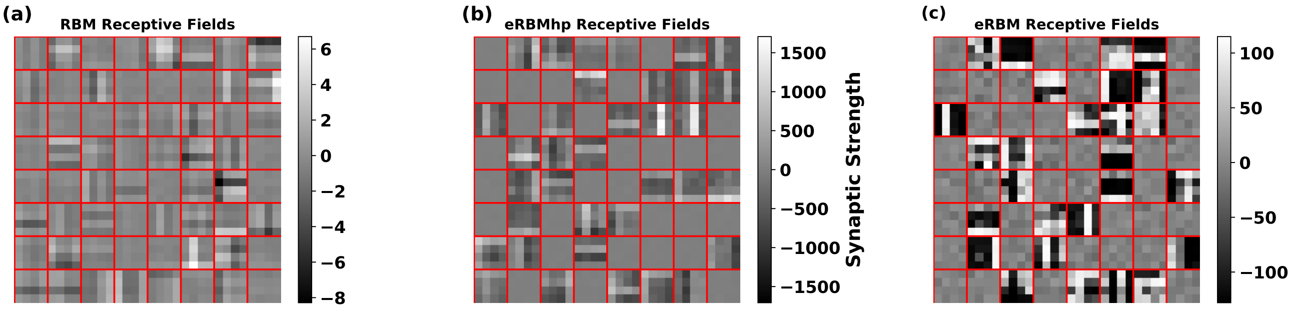

Figure 16. Event-based Restricted Boltzmann Machine (eRBM) receptive fields. (a) Classical RBM of infinite precision trained on the bars and stripes data set, **(b)** hRBMhp with 16-bit precision synaptic weights, and **(c)** eRBM with 8-bit precision synaptic weights.
